# The effect of endplate preselection when measuring supine versus standing cobb angle change in idiopathic scoliosis

**DOI:** 10.1186/1748-7161-10-S1-O43

**Published:** 2015-01-19

**Authors:** Bethany E Keenan, Maree T Izatt, Geoffrey N Askin, Robert D Labrom, Mark J Pearcy, Clayton J Adam

**Affiliations:** 1 QUT/Mater Paediatric Spine Research Group, Institute of Health and Biomedical Innovation, Queensland University of Technology and Mater Health Services, Brisbane, QLD, Australia

## Objectives

The primary aim of this study was to determine whether endplate pre-selection makes a difference to the Cobb Angle change between supine and standing which is known to occur in idiopathic scoliosis. A secondary aim of this study was to identify which (if any) patient characteristics were correlated with supine versus standing Cobb change.

## Methods

Female Adolescent Idiopathic Scoliosis (AIS) patients with right-sided thoracic major curves were included in the retrospective study. Clinically measured Cobb Angles from existing standing coronal radiographs and fulcrum bending radiographs were compared to existing low-dose supine CT scans taken within 3 months of the reference radiograph. Reformatted coronal CT images were used to measure supine Cobb Angle variability with and without endplate pre-selection (end-plates selected on the radiographs used on the CT images). Inter and intra-observer measurement variability was assessed. Multi-linear regression to investigate whether there was a relationship between supine to standing Cobb angle change and eight variables: patient age, mass, standing Cobb angle, Risser sign, ligament laxity, Lenke type, fulcrum flexibility and time delay between radiograph and CT scan.

## Results

**Figure 1:**
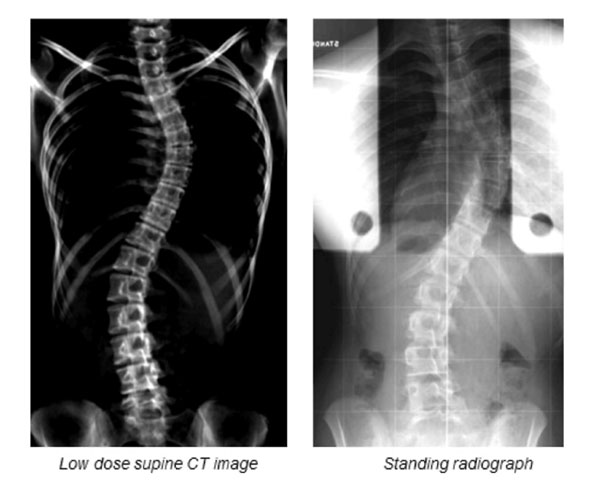
An example of a reformatted supine CT image Cobb 46° (left) and the corresponding standing radiograph Cobb 58° (right)

Fifty-two patients were included, with mean age of 14.6 (SD 1.8) years; all curves were Lenke Type 1 with mean Cobb Angle on supine CT *without* pre-selection of endplates of 41.8° (SD 6.4°) and 51.9° (SD 6.7°) on standing radiographs. The mean Cobb angle on supine CT images *with* endplate pre-selection was 40.5° (SD 6.6). The mean fulcrum bending Cobb Angle for the group was 22.6° (SD 7.5°). The 10° increase from supine to standing is consistent with existing literature. Pre-selecting vertebral endplates was found to increase the mean signed Cobb change by 0.6° (SD 2.3, range -9 to 6)°. When free to do so, observers chose different levels for the end vertebrae in 39% of cases. Multi-linear regression revealed a statistically significant relationship between supine to standing Cobb Angle change with: fulcrum flexibility (p=0.001), age (p=0.027) and standing Cobb Angle (p<0.001). In patients with high fulcrum flexibility scores, the supine to standing Cobb Angle change was as great as 20° (Figure 1). The 95% confidence intervals for intra-observer and inter-observer measurement variability were 3.1° and 3.6°, respectively.

## Conclusions

Pre-selecting vertebral endplates causes minor changes to the mean supine to standing Cobb change. There is a statistically significant relationship between supine to standing Cobb change and fulcrum flexibility such that this difference can be considered a potential alternative measure of spinal flexibility.

